# Complex Regional Pain Syndrome: An update

**DOI:** 10.31138/mjr.30.1.16

**Published:** 2019-03-28

**Authors:** Christina Misidou, Charalampos Papagoras

**Affiliations:** First Department of Internal Medicine, University Hospital of Alexandroupolis, Democritus University of Thrace, Alexandroupolis, Greece

**Keywords:** Complex regional pain syndrome, reflex sympathetic dystrophy, algoneurodystrophy, causalgia, nociceptive pain, neuropathic pain

## Abstract

Complex regional pain syndrome (CRPS) is a perplexing painful syndrome of the extremities usually following a harmful event. It is distinguished in two types, mainly depending on the presence of nerve injury. Although its prevalence may vary depending on social and ethnic factors, middle-aged women seem to suffer most often and the upper limb is the most commonly affected extremity. Apart from pain, which is the dominating feature, the clinical picture unfolds across several domains: sensory, motor, autonomic and trophic. This syndrome develops in two phases, the acute (warm) phase, with the classic symptoms of inflammation, and the chronic (cold) phase, often characterized by trophic changes of the soft tissues and even bones. Although the syndrome has been studied for over two decades, no imaging or laboratory test has been established for the diagnosis and recently proposed diagnostic criteria have not yet been validated and are only occasionally applied. Its pathophysiology is still quite obscure, although the most likely mechanisms involve the classic and neurogenic paths of inflammation mediated by cytokines and neuropeptides, intertwined with changes of the autonomic and central nervous system, psychological mechanisms and, perhaps, autoimmunity. Although plenty of treatment modalities have been tried, none has been proven unequivocally efficacious. Apart from information and education, which should be offered to all patients, the most effective pharmacological treatments seem to be bisphosphonates, glucocorticoids and vasoactive mediators, while physical therapy and rehabilitation therapy also make part of the treatment.

## INTRODUCTION

Complex regional pain syndrome (CRPS) is a rare chronic pain disorder affecting the upper or lower extremities. It is characterized by pain disproportionate to the intensity of the triggering factor and may have a later onset than the time of the harmful event. The syndrome is known to have sensory, motor, autonomic and trophic manifestations, usually lacking a clear dermatomal or peripheral nerve distribution pattern.^1^ The clinical course of the disease varies from mild self-limiting to chronic persistent which may lead to disability.

## CLASSIFICATION

The term Complex Regional Pain Syndrome was introduced in 1994 by the International Association for the Study of Pain (IASP) in the second edition of the Classification of Chronic Pain. The syndrome was divided into CRPS type 1, formerly known as reflex sympathetic dystrophy (RSD), and CRPS type 2, formerly known as causalgia.^2^ While both types occur typically after trauma, the key distinguishing feature is the presence of a definite nerve injury, which is absent in type 1, but present in type 2 CRPS. This distinction is relevant to the nature of chronic pain, which is considered nociceptive in the former and neuropathic in the latter type, although both types may actually represent different parts of a continuous spectrum. A third type (not otherwise-specified) has also been proposed for those patients partially meeting criteria for either type 1 or 2 and lacking a better explanation for their symptoms and signs.^3^

The diagnosis of CRPS is purely a clinical one. However, several efforts have been made to set diagnostic criteria in order to facilitate clinical communication and standardization for research purposes.^4^ The first widely accepted set of criteria was proposed in Orlando and endorsed by IASP in 1994 (*Table 1*).^2^

**Table 1.** 1994 IASP criteria for Complex Regional Pain Syndrome (CRPS)

The presence of a preceding noxious event or immobilization Continuous pain, allodynia or hyperalgesia, disproportional to the supposed inciting event Evidence at any time of swelling, blood flow changes in the skin or sudomotor abnormalities of the affected area Absence of another condition that would explain the symptoms

A patient is diagnosed with CRPS, if criteria 2–4 are met.

Criterion 1 is not mandatory for the diagnosis as 5–10% of patients will have no such history. In the absence of major nerve damage, CRPS type 1 is diagnosed, in the presence of such damage CRPS type 2 is diagnosed instead.

Adapted from [2]

An advantage of these criteria is that they are easy to apply in everyday clinical practice and have a high sensitivity (0.98). However, plenty of studies have shown that their specificity is poor (0.36) and other conditions may mistakenly be considered as CRPS. Indeed, in only 40% of cases diagnosed with CRPS the diagnosis is correct,^5^ while up to 37% of patients with diabetic neuropathy meet criteria for CRPS.^6^ This is probably due to the vague definition of symptoms, which may only be historical, and a degree of subjectiveness in their interpretation (e.g., “disproportionate”). To overcome this, a workshop was held in Budapest in 2003, which resulted in a new, more detailed and likely more specific set of criteria (Budapest criteria, *Table 2*).^7^

**Table 2.** The Budapest criteria for Complex Regional Pain Syndrome (CRPS)

Continuous pain disproportional to the inciting event At least one symptom in ≥3 of the following categories: Sensory (hyperesthesia, allodynia) Vasomotor (temperature asymmetry, skin colour changes, skin colour asymmetry) Sudomotor/Edema (edema, sweating changes, sweating asymmetry) Motor/Trophic (decreased range of motion, weakness, tremor, dystonia, trophic changes affecting the skin, nails, hair) At least one sign present upon evaluation in ≥2 of the following categories Sensory (Evidence of hyperalgesia and/or allodynia) Vasomotor (Evidence of temperature asymmetry and/or skin colour changes/asymmetry Sudomotor/Edema (Evidence of edema and/or sweating changes/asymmetry) Motor/Trophic (Evidence of decreased range of motion and/or weakness, tremor, dystonia and/or trophic changes affecting the skin, nails, hair) Absence of another diagnosis that would better explain the symptoms and signs

A patient is diagnosed with CRPS, if all four criteria are met. For research classification at least one symptom from all four categories and at least 1 sign from all categories should be attested.

Adapted from [7]

## EPIDEMIOLOGY

A population-based study in Olmsted County, Minnesota, over the period 1989–1999, applying the IASP criteria resulted in an incidence rate for CRPS type 1 of 5.46 per 100,000 person-years and a period prevalence of 20.57 per 100,000. On the other hand, CRPS type 2 incidence rate was just 0.82 per 100,000 person-years. The female-to-male ratio was 4:1, while the median age of onset was 46 years.^8^ In a subsequent Dutch study, the incidence of CRPS was estimated at 26.2 per 100,000 person-years, again with a female predominance.^9^ Both studies also showed that the upper extremity is the most commonly affected. In a more recent study from the US, the diagnosis of CRPS emerged at a rate of 70 per 100,000 hospital discharges over a 4-year period.^10^ The most likely causes for those variations (*Table 3*) are differences in the type of source data and the methods of case ascertainment. Indeed, in a literature search aiming to assess the use of the IASP criteria, only 38% of the publications mentioned the application of the criteria and only 15% of the referenced publications satisfied them.^11^ Apart from female gender, other epidemiological risk factors are Caucasian race, high household income and private insurance. It is not yet clear how these factors are related to CRPS and whether there is a biological factor involved. It is suggested that patients with a higher socioeconomic status have access to better healthcare services and a closer follow up. Various comorbidities also seem to affect the occurrence of CRPS, including depression, headache and drug abuse.^10^ In a prospective study by Bean et al. investigating how psychological factors influence the recovery of patients with CRPS, it was concluded that anxiety, pain-related fear and disability have a negative effect, thus implicating psychological parameters for poorer recovery.^12^ In contrast, some diseases do not appear to associate with the syndrome, such as diabetes, anemia, obesity and hypothyroidism.^10^

**Table 3.** Incidence and prevalence of complex region pain syndrome type 1 and 2 across different studies

Author, year (ref)	Country, period	Incidence (per 10^5^/year)	Prevalence (per 10^5^)
Sandroni P, 2003 (8)	Minnesota, 1989–1999	Type 1: 5.46 Type 2: 0.82	Type 1: 20.57 Type 2: 4.2
De Mos M, 2007 (9)	The Netherlands, 1996–2005	26.2	-
Elsharydah A, 2017 (10)	USA, 2007–2011	Rate of CRPS diagnosis: 17.5/10^5^ hospital discharges per year	

## CLINICAL PRESENTATION

Inciting events may be a fracture, particularly distal radius or Colles’ fracture, which has been associated with a CRPS incidence up to 36.7%. Orthopaedic surgery of the extremities has also been associated with the occurrence of CRPS, as well as other types of trauma, immobilization and stroke.^13^

Two phases of the syndrome have been described: first, the acute or “warm” phase, during which the affected limb shows classical signs of inflammation - *calor*, *dolor*, *rubor*, *tumor*.^1^ The symptoms usually appear distally to the area of trauma like a glove or stocking.^14^ Patients describe a constant, deep pain that exacerbates with movement or temperature changes.^15^

The second, chronic or “cold” phase starts about 6 months later, as the inflammation subsides. The quality of the pain is different, more persistent while resting and difficult to treat. Some patients experience muscular spasms.^14^ Atrophies may occur in the skin, subcutaneous tissue and muscles, even local osteoporosis of the underlying bones.^14^ Nail and hair growth are altered, either increased or decreased with quality changes.^15–16^

Autonomic symptoms include hyper- or hypohidrosis and skin colour changes, mainly the limb turning red.^15^ Motor disorders appear in most cases with CRPS: in the initial phase, movement is reduced because of edema and fear of inducing pain with movement (kinesiophobia); in the chronic stage, fibrosis ensues limiting movement.^1^ Although the key distinguishing feature between type 1 and type 2 CRPS is the presence of nerve injury in the latter, the symptoms in type 2 still exceed the territory of the injured nerve and are far more complex than expected for neuropathic pain, resembling, thus, to the symptoms of CRPS type 1.^17^ Besides, in the paper proposing the Budapest criteria, it is questioned whether there is a clinical utility in the differentiation between type 1 and 2, although the distinction was maintained.^7^

## DIAGNOSIS

As no gold-standard tests for CRPS exist, the diagnosis is essentially clinically assisted with the application of proposed criteria.^7^ No serum markers or imaging findings with high diagnostic value have been identified. Plain radiographs may show bone demineralization, although this occurs relatively late and is not specific.^17^ Magnetic resonance imaging (MRI) may show spotted bone-marrow edema, cutaneous edema, joint effusion or contrast uptake in the skin and the synovium. However, these findings have a low overall sensitivity (35%) and a low-moderate predictive value for CRPS, although the specificity is quite high (91%).^18^ Some researchers have proposed the use of three-phase bone scintigraphy (TPBS), which shows alterations in the peri-articular bone metabolism.^19^ This test, particularly phase 3, is highly specific, especially for the upper limb,^18–19^ but when applied in patients with CRPS diagnosed with the Budapest criteria, the sensitivity is quite poor (0.551).^20^ In conjunction with the TPBS imaging, serum osteoprotegerin, an osteoblast activity marker, could be used as a diagnostic tool with a sensitivity about 0.74 and a specificity of 0.8.^21^ There was a significant positive correlation between increased radiotracer uptake in TPBS phase 3 and serum osteoprotegerin in patients with CRPS suggesting an increased osteoblast activity.^21^

Functional brain MRI has been used to investigate the role of the central nervous system in CRPS. Abnormalities in central motor function and changes in cortical paths could be used in the future as possible biomarkers.^22–23^

In conclusion, the diagnosis of CRPS is purely clinical, based on the history, symptoms and signs. Classical radiographs should always be performed to assess bone integrity, given the strong association with trauma. More elaborate studies, such as TPBS and MRI of the affected extremity may assist by revealing findings compatible with CRPS and by excluding other diagnoses, such as synovitis, infections or tumors.

## PATHOPHYSIOLOGY

**Figure 1.**
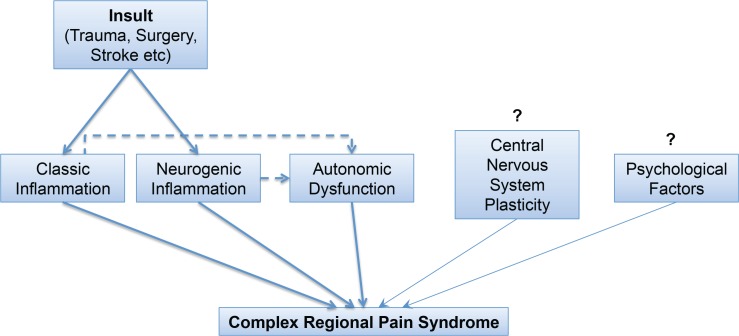
Pathophysiological connections in the complex regional pain syndrome. An insulting event triggers classic and neurogenic inflammation, which are responsible for the inflammatory signs at least of the warm phase and, perhaps, contribute to the autonomic dysfunction. Autonomic dysfunction is also responsible for several aspects of the syndrome, while interventions targeting it have been beneficial, mainly in case series. Central nervous system (CNS) plasticity and psychological factors likely complete the process. However, the exact mechanisms eliciting the CNS changes, linking to the psychological factors and ultimately implicating both of these factors to the clinical presentation of the syndrome are still obscure.

The pathogenesis of the disorder remains obscure. It is considered a multifactorial condition with several contributors: classic inflammation, neurogenic inflammation, impairment of the autonomic nervous system and changes of the central nervous system (CNS) (*Figure 1*).

### Classic inflammation

The acute phase of CRPS implies that classic inflammatory mechanisms are likely involved in its pathophysiology. Tissue trauma is considered to trigger an exaggerated and persistent release of pro-inflammatory cytokines such as tumour necrosis factor-α (TNFα), interleukin (IL) 1β and IL-6, which activate the cascade of inflammation that results in a painful, red and swollen extremity.^13,24^ Indeed, in a systematic review and meta-analysis of studies on mediators of classic inflammation in CRPS, it was found that IL-8 is significantly elevated in the blood during the acute phase, while several other pro-inflammatory mediators, including TNFα, interferon-γ (IFNγ), IL-2, monocyte chemoattractant protein-1 (MCP-1) and bradykinin are raised during the chronic phase. Increased IL-6, MCP-1 and macrophage inflammatory protein 1β (MIP-1β) were also found in the blister fluid from the affected limb.^25^ Although classic inflammation seems more relevant to the warm phase of CRPS, some studies have suggested that even in the cold stage inflammation may play a role, as well.^26^ For instance, it has been shown that the levels of pro-inflammatory cytokines IL-6 and TNF*α* in the affected extremity are comparably disturbed between cold and warm CRPS. A hypothesis might be that in some patients increased vasomotor activity masks a parallel inflammatory process, which is, though, revealed after vasodilatory treatment. Besides, vasoconstriction results from an imbalance of vasodilating (e.g., nitrogen oxide) and vasoconstricting substances (e.g. endothelin-1), which, in turn, may be induced by pro-inflammatory cytokines.^24^

### Neurogenic inflammation

Neurogenic inflammation probably plays a central part in the development of CRPS. It begins with the stimulation of the peripheral endings of the nociceptive C-fibres, which, subsequently, conduct the stimulus not only afferently to the dorsal ganglia, but also efferently through branches extending back into the afflicted tissue. This backward firing results in the release of several pro-inflammatory neuropeptides the most important being substance P, calcitonin gene-related peptide (CGRP) and neurokinin A, as well as others including adrenomedullin, neurokinin B, vasoactive intestinal peptide, neuropeptide Y and gastrin-releasing peptide.^27^ CGRP activates CGRP1 receptor in smooth muscles and endothelial cells inducing vasodilation of arterioles, while substance P and neurokinin A promote vascular permeability by activating neurokinin A1 receptors in endothelial cells.^1,27^ These changes result in hyperemia, tissue edema and exudation of leukocytes. Besides, CGRP and substance P activate resident cells, such as mast and dendritic cells. These, in turn, release inflammatory mediators including histamine, serotonin, TNFα that attract inflammatory cells and further promote inflammation, but also act upon local nociceptive Aδ-fibers inducing peripheral nerve sensitization.^27–28^ Finally, CGRP promotes sweat gland function, as well as hair growth.^1,27^

### Autonomic system

Several factors indicate the role of the sympathetic nervous system in the appearance of CRPS. Those include changes in skin colour, hyper- or hypohidrosis and changes in the extremity temperature.^29^ Systemic manifestations of sympathetic dysfunction have even been observed, such as increased heart rate, reduced heart rate variability and impaired orthostatic response.^30^ In the warm phase of CRPS, local norepinephrine release from sympathetic fibers is decreased leading to increased cutaneous blood flow. However, in the chronic cold phase, α-adrenoreceptors are more sensitive to circulating catecholamines leading to vasoconstriction and decreased blood flow.^24,31^ Expression of adrenoreceptors on nociceptive fibers leads to sympatho-afferent coupling producing sympathetically mediated pain. Indeed, under conditions of sympathetic activation, there is a considerable increase of pain intensity in patients with CRPS.^13,32–33^

### Central Nervous System-Brain Plasticity

Some studies suggest maladaptive neuroplasticity of the motor and somatosensory cortex. In 2000 Birklein et al. published an analysis of 145 cases studying the neurological findings in CRPS. Ninety-seven percent of patients manifested motor dysfunction such as tremor, exaggerated reflexes, dystonia and myoclonic jerks.^34^ The brain areas that seem to participate in the motor dysfunction are the primary motor cortex, supplementary motor cortices and posterior parietal cortices. The reorganization of the central motor circuits is thought to lead to an increased activation of the motor cortex and to motor dysfunction of the affected extremity.^35^ However, a systematic review published in 2013 concluded that there was no significant impairment in the representation of the affected limb on the motor cortex.^36^

Changes in the somatosensory cortex have been investigated by several researchers in order to explain hyperalgesia. The affected body part seems to be represented on a smaller area on the primary somatosensory cortex.^37^ Sensory stimuli on the affected extremity trigger a complex cortical network, including areas of nociceptive, cognitive and motor processing different than the pattern triggered by similar stimuli on the unaffected side. Consequently, the patient does not only feel pain during the movement of the hand or leg, but also with the thought of movement.^38^ Furthermore, it is hypothesized that the continuous nociceptive stimulation induces plastic changes in the motor and somatosensory networks, so that hyperalgesia and chronic pain is not only the consequence of changes in the CNS, but also the cause.^39^

### Psychological Factors

A cross-sectional study comparing psychological factors between patients with CRPS or low back pain showed that in the CRPS group pain intensity correlated with anxiety. Moreover, pain and, particularly, depression were the couple of factors that significantly predicted disability.^40^ Another prospective multicentre study investigated the effect of various psychological factors (agoraphobia, depression, somatization, insufficiency, sensitivity, insomnia, life events) on the risk of CRPS development in 596 patients with a single fracture. Although 7% of patients eventually developed CRPS type 1, none of the psychological scores were significantly different from those of the general population, nor could any psychological factor be identified as a predictor of CRPS.^41^ Similarly, in a systematic review no association between psychological factors and the occurrence of CRPS could be demonstrated, except that patients with more life events were found to carry a higher risk of developing CRPS.^42^

### Autoimmunity

There is evidence that CRPS might involve an autoimmune component, as well. In some cases, IgG autoantibodies with agonist-like properties on the β2-adrenergic and muscarinic-2 receptor were present,^43^ as were autoantibodies against α-1a adrenoreceptors.^44^ Another study by Kohr showed that 30–40% of patients who suffered from CRPS had surface-binding autoantibodies against an autonomic nervous system autoantigen.^45^ The autoimmune nature of CRPS was further upheld by Goebel and his colleagues who performed a randomized, double-blind, placebo-controlled crossover trial of intravenous immunoglobulin (IVIG) including 13 patients with moderate to severe pain due to CRPS. The average pain intensity significantly reduced following IVIG treatment compared to placebo.^46^ However, this result was not replicated in a larger study.^47^

## TREATMENT

As CRPS is a multifactorial syndrome, its management extends over several domains and treatment modalities, such as patient information and education, pharmacological treatments, physical and occupational therapy and psychological support. The aim of treatment is to relieve pain and restore the functionality of the affected limb. Although the course of the disease is variable and there is no strong evidence that it is modified by treatment, therapy should not be delayed, as patients with a more chronic course carry a worse prognosis.

### Patient information and education

As in every chronic disease, patients and their families should be informed about their condition, in order to realize the nature of their symptoms and the course of the disorder.^48^ Education will also allow a more active participation of the patients in the management of their disease by enabling them to form their own understanding of the therapeutic strategy proposed by the physician, to build the knowledge and skills required for the rehabilitation process and to gain trust into this process, improving eventually their adherence to the treatment plan.

### Pharmacological treatments

#### Non-steroidal anti-inflammatory drugs

Non-steroidal anti-inflammatory drugs (NSAIDs) have been used to treat pain and inflammation. However, a randomized double-blind placebo-controlled trial of pare-coxib, 80mg on two consecutive days including only 20 patients with CRPS 1 and 2 of the upper limb, showed no benefit of parecoxib on pain or edema.^49^ Similarly, a randomized trial including 60 patients with CRPS following stroke compared the effectiveness of a one-month treatment with either a tapering dosage of prednisolone starting from 40mg daily or piroxicam 20mg daily. At the end of treatment there was a significant improvement of the CRPS score in patients on prednisolone, but no improvement in those on piroxicam.^50^ Hence, currently there is no evidence supporting the use of NSAIDs for the treatment of CRPS.

#### Glucocorticoids

As classic inflammation is likely involved at least in the early stages of the disease, glucocorticoids might be beneficial. However, relevant studies are heterogeneous as regards CRPS definition, patient characteristics, glucocorticoid formulations and dosages employed, while practically all of them included low numbers of patients. In an early randomized placebo-controlled trial including 23 patients with CRPS type 1, 10 mg of oral prednisone three times daily for up to 12 weeks were proven more effective than placebo in producing clinical improvement.^51^ In another prospective study, treatment of patients with shoulder-hand syndrome post stroke with 32 mg of oral methylprednisolone daily for 14 days followed by a 14-day taper resulted in 31 out of 36 patients being almost asymptomatic within 10 days of treatment.^52^ Moreover, a case series revealed that, in patients with RSD who had failed exercise therapy, long-acting glucocorticoids (80mg of Depo-Medrol administered no more frequent than fortnightly for up to 4 injections) resulted in improvement of limb mobility, pain, swelling and strength.^53^ Finally, in a more recent case series including 31 patients with CRPS, a short course of prednisone with a starting dose of 40–60 mg per day and a quick tapering showed both short- and long-term benefits across various outcomes, such as pain, swelling, mobility, strength and limb functionality.^54^ On the other hand, in a prospective study including 31 patients with CRPS of more than 3 months duration who had failed standard treatments, administration of high-dose prednisolone (60–100mg/day) with a rapid taper-off over 16–22 days showed that the reduction in average pain intensity missed statistical significance, although borderline. This study showed that in chronic CRPS high dose glucocorticoids may not be as effective as in the acute phase, while the associated adverse events should also be taken into account.^55^

#### Bisphosphonates

Bisphosphonates were recently introduced in the treatment of CRPS, although the exact therapeutic mechanism still remains unclear. Plenty of suggestions have been proposed, the most common being through the regulation of inflammatory mediators and the inhibition of proliferation and migration of bone marrow cells.^29^ More importantly, several, mainly small-scale, studies support the effectiveness of bisphosphonates in CRPS. The earliest was a randomized double-blind trial of intravenous alendronate (7.5mg daily for 3 days) or placebo, which showed a significant improvement of pain, tenderness, swelling, and motion.^56^ These results were later confirmed in another double-blind placebo-controlled trial, which used a more convenient dosing regimen of alendronate, 40mg daily per os for 8 weeks. Throughout 12 weeks, significant benefits were observed in patients on alendronate as regards spontaneous pain, pressure tolerance and mobility compared to the control group. In a further 8-week extension of the study, patients continuing alendronate showed additional improvements in the same outcomes. Apart from two patients reporting upper gastrointestinal symptoms, no unexpected adverse events emerged with this increased dosage of alendronate, making it therefore a potentially effective, safe and feasible pharmacological modality for CRPS.^57^

Similar beneficial effects were reported for intravenous clodronate,^58^ intravenous neridronate,^59^ and intravenous pamidronate,^60–61^ all types of bisphosphonates, though, not widely available. Notably, a randomized study comparing intravenous pamidronate (three infusions of 60mg each) to oral prednisolone (1mg/kg tapered off over 14 days) in CRPS post stroke showed that pamidronate was as effective as prednisolone in pain control.^61^

#### Calcitonin

Calcitonin is thought to reduce pain in CRPS through release of β-endorphin in CNS and inhibition of bone resorption.^62^ In an early study, Gobelet et al. randomized 24 patients with RSD undergoing physical therapy to additional subcutaneous calcitonin 100 mg daily for 3 weeks, or no additional treatment and showed that the group on calcitonin had more rapid pain relief.^63^ The same group subsequently performed a similar study with intranasal calcitonin (300UI daily) and background physical therapy showing greater improvement of pain, range of motion and the ability to work with calcitonin compared to placebo.^64^ However, another prospective randomized double-blind study demonstrated no effect of intranasal calcitonin (400UI daily) on clinical progression of CRPS.^65^ Finally, other investigators showed that calcitonin was no more effective than paracetamol, both administered to patients undergoing physical therapy.^66^ In conclusion, the scientific evidence supporting the use of calcitonin for the treatment of CRPS is rather weak and, given the recent safety concerns regarding its use, it is no longer recommended.^67^

#### Free radical scavengers

Therapy of CRPS with free radical scavengers is based on the hypothesis that in CRPS toxic oxygen and hydroxyl free radicals are overproduced.^62^ Three free radical scavengers have been used so far: dimethylsulfoxide (DMSO), N-acetylcysteine (NAC) and mannitol. DMSO 50% cream was applied locally to patients with acute CRPS in combination with physiotherapy. After 2 months, the visual analogue score for pain and RSD scores were improved.^68^ When DMSO 50% (5 times per day) was compared to NAC (600 mg 3 times per day), DMSO treatment was more efficacious for warm CRPS type 1 and for treating dysfunctions of the lower extremity, while NAC was more effective for cold CRPS type 1.^69^ Moreover, DMSO 50% cream seems favourable compared to ismelin block treatment for CRPS in early stages.^70^ Regarding mannitol, a randomized double-blind placebo-controlled trial showed no significant benefit of mannitol 10% infusions compared to placebo in patients with CRPS type 1.^71^

#### Vitamin C

Vitamin C has been used for the prevention of CRPS based on the concept that it inhibits local inflammatory pathways via antioxidant mechanisms.^46^ A meta-analysis of three randomized placebo-controlled trials showed that 500 mg of vitamin C per day for 50 days decreases the one-year risk of CRPS after wrist fracture.^72^

#### Opioids

Although opioids constitute the second-line treatment for neuropathic pain, their effectiveness is not yet established in CRPS and their multiple side effects limit their use.^13^

#### Anticonvulsants

Anticonvulsants, such as gabapentin and pregabalin, have been proven to relieve pain in some neuropathic pain syndromes. With this assumption, Van de Vusse et al. conducted a randomized placebo-controlled crossover trial using gabapentin to treat CRPS type 1. They concluded that the pain relief was not significant with gabapentin, but there was a greater reduction of the sensory deficit in the affected extremity.^73^ There is still no evidence on the effectiveness of pregabalin, carbamazepine or phenytoin in CRPS.

#### Vasoactive mediators

Sympatholytic drugs have been tried in patients with CRPS, in order to inhibit the sympathetically mediated pain.^13^ The α-sympathetic blocker phenoxybenzamine has shown positive results in reducing pain in the acute stage.^74–75^ Clonidine, an α2-adrenergic agonist, administered locally via a transdermal patch, has also been shown to be efficacious in treating hyperalgesia.^76^ The effectiveness of phenoxybenzamine, as well as nifedipine, a calcium-channel blocker, in the treatment of CRPS was reported in a series of 59 patients.^77^ Both treatments produced beneficial effects, when administered in the early stages of the syndrome, but their effectiveness was less evident, when the treatment started in the chronic stage.

In conclusion, although plenty of pharmacological treatments have been tried for the treatment of CRPS, the published studies are mainly underpowered and heterogeneous in terms of patient characteristics and drug dosages. Therefore, safe conclusions are difficult to draw. A recent network meta-analysis focusing on pain management in CRPS concluded that for CRPS with symptom duration less than 12 months the best treatment are bisphosphonates followed (by far) by glucocorticoids. For CRPS with symptom duration of at least 12 months the best treatment was calcitonin followed by bisphosphonates and vasodilators.^78^

### Physical Rehabilitation

Physical and occupational therapy, with or without medical therapy, are first-line treatments of CRPS in order to avoid kinesiophobia. Although initiation of physiotherapy in the early stages has been proven more beneficial, it may also help in chronic CRPS.^79^ Physical therapy is more efficacious in reducing pain and improving active mobility,^80^ while use of the affected limb in daily activities is improved through occupational therapy.^13^ Given that neuroplasticity is an important part of the CRPS pathophysiology, graded motor imagery is one of the most beneficial tools of physical therapy for reducing pain.^81^ Particularly, mirror therapy, which has been used to reduce the pain of amputated ghost limb, is beneficial in alleviating pain and improving functionality in CRPS as well. Having a mirror placed between the healthy and the affected limb, the patient is encouraged to move the healthy limb in front of the mirror and at the same time watch the movement through the mirror. In this way, an illusion is created that the affected limb is also moving, although without feeling pain. Several studies gave positive results using this cost-free and easily applied therapeutic method.^82^

### Invasive treatments

When medication has failed to relieve pain in CRPS, several invasive procedures have been employed, such as sympathetic blockade, surgical sympathectomy, spinal cord stimulation and even amputation. However, research is still required, in order to assess their cost-benefit balance and their indications and place in the treatment of CRPS.^79^

## CONCLUSION

Complex regional pain syndrome is a chronic and multifactorial pain syndrome, emerging after trauma and causing various degrees of disability. Inflammation, both classic and neurogenic, plays a major role in the pathogenesis of the disease, coupled with disorders of the autonomic nervous system, CNS plasticity and, possibly, psychological factors. As there is no objective test, the diagnosis is reached on clinical grounds. The treatment is multidisciplinary, involving patient education, medications (bisphosphonates, glucocorticoids, gabapentin, topical antioxidants, etc.), physical and occupational therapy. More research is needed in order to elucidate its pathogenesis, which will allow the design of targeted treatments to be tried in large prospective studies.

## Abbreviations

CGRP: Calcitonin gene-related peptide
CNS: Central nervous system
CRPS: Complex regional pain syndrome
DMSO: Dimethylsulfoxide
IASP: International Association for the Study of Pain
IFN: Interferon
IL: Interleukin
IVIG: Intravenous immunoglobulin
MCP: Monocyte chemoattractant protein
MIP: Macrophage inflammatory protein
MRI: Magnetic resonance imaging
NAC: N-acetylcysteine
NSAIDs: Non-steroidal anti-inflammatory drugs
RSD: Reflex sympathetic dystrophy
TNF: Tumor necrosis factor
TPBS: Three-phase bone scintigraphy
